# Supplementary material for: Real-World Study on Effectiveness of Molnupiravir and Nirmatrelvir–Ritonavir in Unvaccinated Patients with Chronic Respiratory Diseases with Confirmed SARS-CoV-2 Infection Managed in Out-Patient Setting
Source: Viruses. 2023 Feb 23;15(3):610. doi: 10.3390/v15030610 (PMC10055981; doi:10.3390/v15030610)
Supplement: Supplementary file 1 [file viruses-15-00610-s001.zip › viruses-2148323-supplementary.pdf]

**Supplementary file**

**Table S1.** Demographic and clinical characteristics of included patients.

| **Variable**[a] | **No anti-viral treatment (n = 2387)** | **NMV-r or MOV (n = 880)** | **p-value** |
|---|---|---|---|
| Age (years), Median [IQR] | 82 [73 – 89] | 83 [73 – 89] | 0.046 |
| Male | 1762 (73.8%) | 579 (65.8%) | <0.001 |
| Ethnic group | | | |
| Chinese | 2363 (99.0%) | 873 (99.2%) | 0.802 |
| Caucasian | 4 (0.2%) | 0 (0%) | |
| Southeast Asian | 6 (0.3%) | 2 (0.2%) | |
| South Asian | 9 (0.4%) | 3 (0.3%) | |
| Japanese | 1 (0%) | 1 (0.1%) | |
| Others | 4 (0.2%) | 1 (0.1%) | |
| Respiratory diseases | | | <0.001 |
| Asthma | 474 (19.9%) | 211 (24.0%) | |
| Bronchiectasis | 358 (15.0%) | 157 (18.1%) | |
| COPD | 1555 (65.1%) | 510 (58.0%) | |
| CCI, Median [IQR] | 1 [1-2] | 0 [0-1] | 0.003 |
| Medication | | | |
| Statin | 1040 (43.6%) | 436 (49.5%) | 0.002 |
| Anti-epileptic | 28 (1.2%) | 13 (1.5%) | 0.488 |
| DOACs<br>Calcium channel blocker | 191 (8.0%)<br>1294 (54.2%) | 67 (7.6%)<br>497 (56.5) | 0.715<br>0.248 |
| Baseline blood test, Median [IQR] | | | |
| Leucocyte count[b] | 6.08 [4.90 – 7.38] | 6.01 [4.91 – 7.40] | 0.437 |
| Neutrophil count[b] | 3.86 [2.93 – 4.92] | 3.90 [3.07 – 4.93] | 0.448 |
| Lymphocyte count[b] | 0.82 [0.50 – 1.32] | 0.94 [0.58 – 1.47] | 0.046 |
| Eosinophil count[b] | 0.04 [ 0.00 – 0.14] | 0.05 [0.00 – 0.14] | 0.210 |
| Estimated GFR, mL/min/1.73m$^2$ | 58.2 [40.5 – 77.0] | 59.6 [38.8 – 77.0] | 0.360 |
| ALT (unit/L) | 12.0 [8.0 – 16.6] | 12.9 [8.8 – 18.0] | 0.107 |
| COVID-related outcomes | | | |
| Hospitalization | 228 (9.6%) | 59 (6.7%) | 0.011 |
| Respiratory failure | 209 (8.8%) | 36 (4.1%) | 0.068 |
| Severe respiratory failure | 91 (3.8%) | 19 (2.2%) | 0.020 |
| Mortality | 120 (5.0%) | 19 (2.2%) | <0.001 |
| Length of stay (days), Median [IQR] | 9 [4-16] | 9 [3-21] | 0.135 |

ALT, Alanine aminotransferase; CCI, Charlson co-morbidity index; COPD, Chronic obstructive pulmonary disease; DOACs, direct-acting oral anticoagulants; GFR, glomerular filtration rate; IQR, interquartile range; MOV, Molnupiravir; m, Meter; min, minute; mL, millilitre; NMV-r, nirmatrelvir-ritonavir; continuous variables are compared by unpaired t-test for continuous variables, categorical variables are compared by $\chi^2$ test

**Table S2.** Risk ratio (RR) for disease outcomes in univariate and multivariate log-binomial regression

| | No. of patients (% in group) | | | RR (95% CI)[#] (Compared with no treatment) | | Adjusted RR (95% CI)[a#] (Compared with no treatment) | |
|---|---|---|---|---|---|---|---|
| **COVID-related outcomes** | **No anti-viral treatment (n = 2387)** | **NMV-r (n = 302)** | **MOV (n = 578)** | **NMV-r** | **MOV** | **NMV-r** | **MOV** |
| Hospitalization | 228 (9.6%) | 15 (5.0%) | 44 (7.6%) | 0.52 (0.31 – 0.87) p=0.01* | 0.80 (0.59 – 1.09) p=0.15 | 0.56 (0.31 – 1.02) p=0.06 | 0.78 (0.55 –1.11) p=0.16 |
| Respiratory failure | 209 (8.8%) | 10 (3.3%) | 40 (6.9%) | 0.38 (0.20 – 0.71) p <0.01* | 0.79 (0.57 – 1.10) p=0.16 | 0.33 (0.15 – 0.74) p <0.01* | 0.74 (0.51 – 1.06) p=0.10 |
| Severe respiratory failure | 91 (3.8%) | 3 (1.0%) | 16 (2.8%) | 0.26 (0.08 – 0.82) p=0.02* | 0.73 (0.43 – 1.23) p=0.23 | 0.23 (0.06 –0.93) p=0.04* | 0.52 (0.27 – 0.99) p=0.048* |
| Mortality | 120 (5.0%) | 4 (1.3%) | 15 (2.6%) | 0.26 (0.01 – 0.71) p=0.008* | 0.52 (0.30 – 0.88) p=0.01* | 0.37 (0.14 –1.01) p=0.051 | 0.42 (0.23 – 0.77) p<0.01* |

*p <0.05 indicated by box shading.
CI, Confidence interval; MOV, Molnupiravir; NMV-r, nirmatrelvir-ritonavir; RR, Risk ratio
[a]Adjusted for age, gender, underlying respiratory disease, baseline estimated glomerular filtration rate, and Charlson co-morbidity index.
Boxes highlighted in blue represent statistical significant findings

**Table S3.** Odds ratio (OR) for disease outcomes in univariate and multivariate logistic regression in sensitivity analysis

| | **No. of patients (% in group)** | | | **OR (95% CI) (Compared with no treatment)** | | **Adjusted OR (95% CI)[a] (Compared with no treatment)** | |
|---|---|---|---|---|---|---|---|
| **COVID-related outcomes** | **No anti-viral treatment (n = 2387)** | **NMV-r (n = 302)** | **MOV (n = 578)** | **NMV-r** | **MOV** | **NMV-r** | **MOV** |
| Hospitalization | 228 (9.6%) | 15 (5.0%) | 44 (7.6%) | 0.50 (0.29 – 0.85) p=0.01* | 0.78 (0.56 – 1.09) p=0.15 | 0.54 (0.28 – 1.01) p=0.05 | 0.76 (0.52 – 1.12) p=0.16 |
| Respiratory failure | 209 (8.8%) | 10 (3.3%) | 40 (6.9%) | 0.45 (0.22 – 0.93) p=0.03* | 0.84 (0.56 – 1.28) p=0.42 | 0.31 (0.14 – 0.71) p <0.01* | 0.72 (0.48 – 1.07) p=0.10 |
| Severe respiratory failure | 91 (3.8%) | 3 (1.0%) | 16 (2.8%) | 0.25 (0.08 – 0.81) p=0.02* | 0.72 (0.42 – 1.23) p=0.23 | 0.22 (0.05 – 0.91) p=0.04* | 0.51 (0.26 – 0.99) p=0.05* |
| Mortality | 120 (5.0%) | 4 (1.3%) | 15 (2.6%) | 0.25 (0.09 – 0.69) p <0.01* | 0.50 (0.29 – 0.87) p=0.01* | 0.36 (0.13– 0.99) p=0.05* | 0.40 (0.21 – 0.76) p<0.01* |

*p <0.05 indicated by box shadingCI, Confidence interval; MOV, Molnupiravir; NMV-r, nirmatrelvir-ritonavir; OR, Odds ratio
[a]Adjusted for age, gender, underlying respiratory disease, baseline estimated glomerular filtration rate, and Charlson co-morbidity index.
Boxes highlighted in blue represent statistical significant findings

**Table S4.** Anti-viral effectiveness in subgroup as measured by adjusted odds ratios with logistic regression in sensitivity analysis

| **COVID-related outcomes** | **No. of patients (% in group)** | | | **Anti-viral effectiveness (95% CI) (Compared with no treatment)** | |
|---|---|---|---|---|---|
| | **No anti-viral treatment (n = 2387)** | **NMV-r (n = 302)** | **MOV (n = 578)** | **NMV-r** | **MOV** |
| Hospitalization | 228 (9.6%) | 15 (5.0%) | 44 (7.6%) | 46.5% (-0.8 – 71.6%) p=0.053 | 23.7% (-11.6 – 47.9%) p=0.163 |
| Respiratory failure | 209 (8.8%) | 10 (3.3%) | 40 (6.9%) | 69.1% (28.9 – 86.5%) p=0.006* | 28.5% (-6.7 – 52.1%) p=0.101 |
| Severe respiratory failure | 91 (3.8%) | 3 (1.0%) | 16 (2.8%) | 77.9% (8.8 – 94.6%) p=0.037* | 49.2% (0.8 – 74.0%) p=0.047* |
| Mortality | 120 (5.0%) | 4 (1.3%) | 15 (2.6%) | 64.2% (1.1 – 87.0%) p=0.048* | 59.9% (24.5 – 78.7%) p=0.005* |

*p <0.05 indicated by box shading.

CI, Confidence interval; MOV, Molnupiravir; NMV-r, nirmatrelvir-ritonavir

Anti-viral effectiveness was calculated the formula, (1-adjusted odds ratio) x 100. The odd ratios were adjusted for age, gender underlying respiratory disease and Charlson co-morbidity index.

Boxes highlighted in blue represent statistical significant findings
